# rNAV 2.0: a visualization tool for bacterial sRNA-mediated regulatory networks mining

**DOI:** 10.1186/s12859-017-1598-8

**Published:** 2017-03-23

**Authors:** Romain Bourqui, Isabelle Dutour, Jonathan Dubois, William Benchimol, Patricia Thébault

**Affiliations:** LaBRI, CNRS UMR5800, Université de Bordeaux, 351, cours de la Libération, Talence cedex, F-33405 France

**Keywords:** sRNA-mediated regulatory network, Bacterial sRNA, Visualization and bioinformatics

## Abstract

**Background:**

Bacterial sRNA-mediated regulatory networks has been introduced as a powerful way to analyze the fast rewiring capabilities of a bacteria in response to changing environmental conditions. The identification of mRNA targets of bacterial sRNAs is essential to investigate their functional activities. However, this step remains challenging with the lack of knowledge of the topological and biological constraints behind the formation of sRNA-mRNA duplexes. Even with the most sophisticated bioinformatics target prediction tools, the large proportion of false predictions may be prohibitive for further analyses. To deal with this issue, sRNA target analyses can be carried out from the resulting gene lists given by RNA-SEQ experiments when available. However, the number of resulting target candidates may be still huge and cannot be easily interpreted by domain experts who need to confront various biological features to prioritize the target candidates. Therefore, novel strategies have to be carried out to improve the specificity of computational prediction results, before proposing new candidates for an expensive experimental validation stage.

**Result:**

To address this issue, we propose a new visualization tool *rNAV 2.0*, for detecting and filtering bacterial sRNA targets for regulatory networks. *rNAV* is designed to cope with a variety of biological constraints, including the gene annotations, the conserved regions of interaction or specific patterns of regulation. Depending on the application, these constraints can be variously combined to analyze the target candidates, prioritized for instance by a known conserved interaction region, or because of a common function.

**Conclusion:**

The standalone application implements a set of known algorithms and interaction techniques, and applies them to the new problem of identifying reasonable sRNA target candidates.

**Electronic supplementary material:**

The online version of this article (doi:10.1186/s12859-017-1598-8) contains supplementary material, which is available to authorized users.

## Background

Bacterial small RNAs (sRNAs) are crucial regulators that often act to transmit environmental signals when bacteria meet stressful growth conditions [1–3]. These sRNAs are known to regulate changes in cell behavior (including cellular metabolism [4] and quorum sensing [5], for instance, to improve the probability for survival. Although some bacterial RNA regulators were identified since the early 80’s, their involvement in numerous physiological responses and the idea of their probable universal distribution in the prokaryotic world have only emerged recently [1]. These findings are deeply modifying our view on the way bacteria can regulate gene expression to rapidly adapt their metabolism in response to environmental changes and/or stresses [4, 6]. The important role of sRNAs, acting as regulators, in the establishment of virulence has been also noted in several bacterial pathogens [6, 7]. Moreover, recent data from high throughput technologies have shown that small RNAs are much more represented in the bacterial world than previously expected [7–9].

Focusing on sRNAs acting as negative or positive post-transcriptional regulators by base-pairing mRNAs, the identification of their targets is challenging and needs a better understanding of the topological and biological constraints behind the formation of sRNA-mRNA interactions. Moreover sRNA can have several targets, and some mRNAs may be hybridized by several sRNAs forming SIM (Single-Input Module) or DOR (Dense Overlapping Regulon) motifs of regulation [10].

In the omics era where the amount of available data is still increasing, one of the challenge facing the use of predictive bioinformatics tools is to be able to make use of the huge number of predicted results. Even with the most sophisticated bioinformatics target prediction tool, the large proportion of false predictions may be prohibitive to enable further analysis. To deal with this issue, sRNA target analysis can be run from the resulting gene lists given by RNA-SEQ experiments when available. However, the number of resulting target candidates may be still huge and cannot be easily interpreted by biologist experts who needs to confront various biological features to prioritize the target candidates.

Pathway analysis tools intend to deal with these issues. Accordingly, one of the most famous software, Cytoscape [11], supports many features dedicated to the visualization and exploration of large networks. The addition of plugins to Cytoscape is also needed for integrating network-dedicated functionalities. For instance, the ReNE plugin [12] enables to query biological databases for building a regulatory network while integrating external heterogeneous data. While transcription factors and microRNAs can be analyzed together, the main limitation lies in the fact that ReNE only supports the analysis of two eukaryotic organisms. In the annotation of gene set using enrichment strategies, the most popular plugins are Bingo and ClueGO [13, 14]. At the moment no integrated tool provides functionalities dedicated to small RNAs to support their analysis by making use of annotations together with interaction and regulatory motif information. However, these issues need to be further investigated.

In this context, we introduce the *rNAV 2.0* software, the first available release of the prototype presented in [15] as a proof of concept (for a comparison of its functionnalities with in addition Cytoscape, see Additional file 1). Our software aims at proposing original facilities, using a combined strategy based on bioinformatics and visualization approaches.

Combining bioinformatics and visualization help biologists in the analysis of such sRNA-mediated regulatory networks by supporting the exploration and visualization of the huge number of predicted sRNA targets produced by existing bioinformatics tools [16].

## Implementation

Information Visualization has now been established as a fruitful strategy to tackle the problem posed by the abundance of information (for an overview on biological data visualization, the reader can refer to [16, 17]).

Main aspect a visualization system, such as *rNAV 2.0*, has to deal with is the representation of the entire network as it eases the identification of the main trends in the data and therefore to guide the user during his/her exploration and to focus his/her attention on interesting parts.

### Technical aspects

*rNAV 2.0* have been mostly developed in C++ but also in Python and is provided under LGPL. The software is based on Tulip framework [18]. Tulip is an information visualization framework for the analysis and the visualization of relational which provides a complete library for the design of interactive visualization applications. The framework also enables the development of plugins to integrate new algorithms, visual encodings, interaction tools and also domain-specific visualizations. Following Munzner’s recommendation [19], Tulip also supports the implementation of user interface overlays and domain specific software.

The *Model-View-Controller* architecture of Tulip allows *rNAV* to support multiple and synchronized views. Any interaction on a view (e.g. selection of an element) implies the automatic update of all views displaying this data.

### Network prediction and functional annotation

The sRNA-mediated regulation network is modelized by a bipartite graph where nodes represent either sRNAs or mRNAs and interactions are given by edges linking an RNA to another.

To predict interactions between sRNAs (fasta file) and mRNAs (embl/genbank file), *rNAV* integrates two well-known bioinformatics tools: ssearch [20], by reversing and complementing the second RNA to focus on interaction instead of homology, and IntaRNA [21]. The functional annotation of sRNA targets is achieved by DAVID webservice [22], a single gene-term enrichment analysis software which exploits several biological knowledge databases. (e.g. GO, KEGG Pathways, UniProt Sequence Features). DAVID computes, for a given sRNA and its putative mRNA targets, the over-represented biological annotations (as well as a significance score) that are associated to the putative sRNA-mRNA interactions.

### Network visualization

**Fig. 1:**
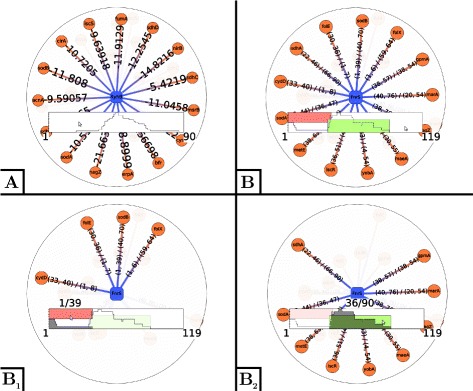
Visualization of the validated targets of the RyhB (panel **a**) and FnrS (panel **b**) small RNAs. For each base of one RNA, a curve is displayed to show the number of interactions each of its bases is involved in. The position clustering algorithm was applied to the RyhB and graph. Two position clusters were calculated and are displayed in *pink* for the positions 1 to 39 and in *green* for the positions 36 to 90. The targets interacting with each position cluster can be selected on demand (see *B*_1_ and *B*_2_)

Several techniques can be considered to visualize networks. Among them, the main options are *matrix based diagrams* (which is merely a visual representation of the adjacency matrix) and *node-link diagrams* (where nodes are represented by a glyph and an edge by a link between the corresponding nodes). While matrix based diagrams are particularly useful for the visual exploration of large and dense graphs as it allows to better perceive local densities, it does not seem to be the best option in the case of sRNA-mediated regulatory networks. Indeed, it would be difficult to emphasize edge’s attributes (e.g. interacting regions, p-values, functional annotations, etc) with such a visualization technique. In *rNAV*, node-link diagrams have therefore been preferred over matrix based diagrams as links between nodes provide a relatively large area to show edge’s attributes (see Fig. 1). To emphasize the bipartite structure of the network, i.e. to help the identification of sRNAs and mRNAs, different colors and shapes are used (in Fig. 1, sRNAs are drawn as blue squircles and mRNAs as orange circles). In addition, edge’s attributes can be displayed along it (e.g. in Fig. 1, putative interacting regions are displayed).

Finally, *rNAV* supports multiple and linked views so as to enable the user to compare the results of different algorithms and/or different algorithms’ settings. In other words, any interaction on a view will affect the others. For instance, selecting elements in one view will also select these elements in all the views (if present in the corresponding networks).

### Available algorithms

To guide the user in his/her exploration, *rNAV* integrates four categories of algorithms: *Filter*, *Coloration*, *Layout* and *Calculation*. While algorithms of the *Coloration* and *Layout* categories respectively allow to color RNAs and putative interactions according to some measures and to lay the network out in the plan, *Filter* and *Calculation* categories contain algorithms to reduce the scope of the analysis. Algorithms of these categories allow to focus the exploration on sub-parts of the network and therefore facilitate the understanding of biological processes and the investigation of the putative cellular organization. Using *Filter* algorithms, the user can specify RNAs of interest according to biological knowledge (e.g. RNA names or identifiers, or regions of the primary sequences), but also according to topological motifs they belong to (e.g. SIM and DOR motifs [10]). Algorithms of the *Calculation* category allow to compute simple however useful graph theoretical measures (e.g. degree or betweenness centrality). In addition that category includes algorithms to cluster for each RNA its putative interactions. That clustering algorithms group putative interactions according to some distance between them. *rNAV* actually integrates two distances measuring either how close their interacting regions are or how similar their functional annotations are. Then using the distance, the MCL clustering [23] is performed to group similar interactions. On the one hand, the interacting regions based distance helps to identify interactions happening on similar regions of the considered RNA, and then functional annotations of each group can be analyzed to identify clusters related to particular biological processes. On the other hand, functional annotations based distance allows to perform the dual analysis. Clustering allows to first group interactions with similar annotations and then identify whether or not a biological process is related to a particular region of the sequence.

To extract interesting candidates from the list of all the putative interactions, the user often needs to perform several and successive filters. *rNAV* supports the edition of algorithm pipelines as an ordered sequence of algorithms of any category. In that sequence, the output of an algorithm is the input of the next one. For a reproducibility purpose, our tool also supports saving of algorithms pipelines. These saved pipelines are then reachable from the *Action list* button in the user interface.

### Interaction tools

*rNAV* integrates classical *zoom and pan* and *box-selection* interaction tools as well as a dedicated functionality that displays on demand detailed informations about an RNA of interest (see Fig. 1). The latter interaction tool has been adapted from [24] to highlight in the visualization the putative interactions of a given RNA. It also shows the number of interactions each base of its primary sequence is involved in with a curve inside. In case where interactions have been clustered, the resulting clusters are displayed as colored rectangles spanning the region of the corresponding interactions (see Fig. 1 *B*_1_ and *B*_2_). These rectangle as well as box-selection can also be used to select neighbors of the focused RNA.

### Exploration awareness

**Fig. 2:**
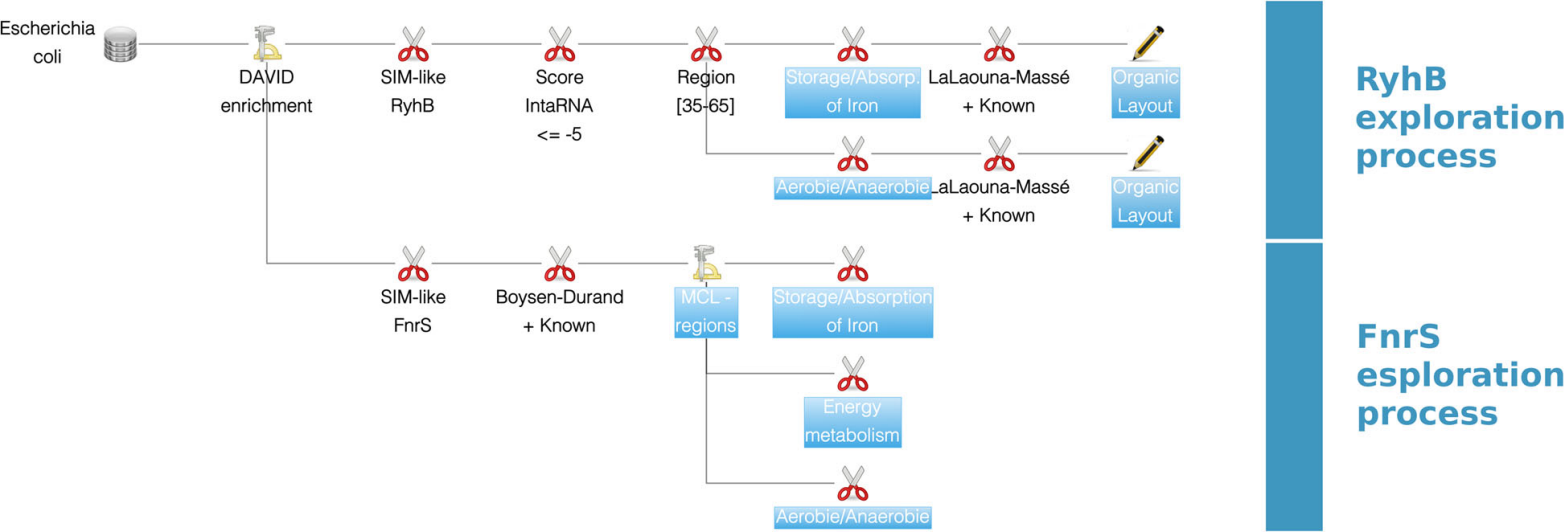
rNAV exploration tree view. This view represents the analytical strategy to explore the sub-networks, which involve RyhB and FnrS sRNAs. Surrounded nodes of the exploration tree correspond to the four views of Figs. 3 and 4

The exploration of the entire network can be time-consuming and may even require multiple analysis sessions. To help the user to remember his/her previous analyses, a combination of features have been integrated in *rNAV*. First, *rNAV* integrates an *exploration tree view* displaying the algorithms that have been run during the analysis (see Fig. 2). In that tree, each node corresponds to a state of the analysis. In particular, the root node corresponds to the entire network. Each time an algorithm is run on a sub-network associated to a node u of the tree, we add a new child to u corresponding to the output of the algorithm. When running a pipeline of algorithms, a new branch is created in the tree where each node of the branch corresponds to an algorithm of the pipeline. The user can also add additional information to each node of the exploration tree, i.e. to each state of the exploration (by default, the information is the name of the corresponding algorithm). Second, the user can save the current state of his/her analysis. In particular, *rNAV* can save the network as well as all the exploration tree. During the next sessions, the user will thus be able to access each state of his/her previous analysis.

## Case applications

These past few years, the rapid and successful evolution of high-throughput sequencing technology has provided an exponential growth of the number of known sRNAs in bacteria. The present estimation of sRNAs known to be expressed by either *Escherichia coli* and/or *Salmonella enterica* is around 300 [25]. Among them, a large set of sRNAs are already known as regulators and are trans-acting with mRNAs by base-pairing with 5’ UTR mRNA regions (see sRNATarBase 3 [26] and EcoCyc [27]). As previously mentioned, sRNA regulation are inter-twisted with transcription factor regulation. One illustration can be given by the mechanisms that lead to the regulation of the iron storage in *Escherichia coli*. Iron is both essential and toxic to most bacteria cells. To survive, the cell has to maintain accurate its rate of iron by rapidly managing the incomes and outcomes of iron. In *Escherichia coli*, the iron rate is mainly regulated both by the FUR transcription factor and the RyhB sRNA.

In addition, the regulation can be coordinated by several sRNAs in response to crossed perturbations of the cell. Again, an illustration can be given with the RyhB and FnrS sRNAs which share four mRNA validated targets. These four genes are involved in different metabolism processes, covering the energetic metabolism (sdhA, sodB) and the response to external stress (marA and nagZ). To go further and analyze the sRNA regulation using *rNAV*, we focused on these two sRNAs, FnrS and RyhB. In this context, we performed an analytical strategy with the objective to propose new target candidates. In our strategy, we exploit the information that can be collected from the validated targets to prioritize the resulting gene lists given by the high-throughput sequencing results of sRNA mutants.

### Analysis of experimental data

To initiate this work, we first constructed the network using *rNAV*, derived from both a multi-fasta file for the sRNA sequences and an embl file for the *Escherichia coli* genome, using adjustable parameters for the 5’ UTR extraction of each mRNA sequences. Interactions were computed using IntaRNA [21] through *rNAV* (see Additional file 2). In each gene/mRNA subset related to a given sRNA, the enrichment of genes having an annotation of interest was performed using a statistical test computed by the DAVID web-services [22].

At the start of this study, the experimental validated targets of these two sRNAs were compiled using the sRNATarBase 3 [26], EcoCyc [27] and [28, 29] (see Table 1) and depicted using *rNAV* in the Fig. 1. The main known information about these two sRNAs is summarized in the following paragraphs and has served as a basis in our analysis:

**Table 1:** List of the experimental validated mRNA targets for the RyhB and FnrS sRNAs versus the results given by rNAV

		sRNATarBase	BSRD	Wright et al.	ECOCYC	rNAV
		3.0 [26]	[42]	[28, 29]	[27]	
RyhB targets	iscS	x	x	x	x	x
	fur		x	x		
	sdhD	x	x			x
	**sodB** ^a^	x	x	x	x	x
	acnA	x	x			x
	fumA	x	x	x	x	x
	nirB	x		x	x	x
	marA	x		x	x	
	bfr		x			x
	nagZ	x		x		x
	ftnA	x	x			
	sdhA	x		x		x
	erpA	x		x	x	x
	sdhC	x		x	x	x
	sdhCDAB			x		x
	cysE	x		x	x	x
	msrB=yeaA			x		x
	hemB				x	
	hemH				x	
	uof				x	
	sodA				x	x
	shiA	x	x		x	
FnrS targets	folX	x	x	x	x	x
	gpmA	x	x	x	x	x
	folE	x	x	x	x	x
	maeA	x	x	x	x	x
	iscR	x		x	x	x
	marA	x		x	x	x
	nagZ	x		x		x
	sdhA	x		x	x	x
	sodA	x	x	x	x	x
	**sodB** ^a^	x	x	x	x	x
	metE	x	x	x	x	x
	cydD	x	x	x	x	x
	yobA	x	x	x	x	x

^a^indicates the shared targets by the two sRNAs

RyhB is a sRNA of 90 nucleotides in length, whose expression is induced by a low level of iron in the bacterium. The analysis of RyhB was motivated by the fact that this sRNA is one of the most studied because of its involvement as a master key in the regulation of iron homeostasis [30]. Accordingly, RyhB was widely experimentally investigated by [31, 32]. Most of the bacteria need to secure sufficient stocks of iron to enable their assessment through essential pathways. As a consequence, numerous proteins are involved to maintain this homeostasis and need to be regulated by sRNAs like RyhB in a precise and coordinated way. Therefore, deciphering its potential action is of great interest in the study of the potentiality of sRNA regulation in complex regulatory networks. Moreover, other of its known targets are involved in the TCA [33]. No experimental work has been done to investigate the region which interacts with the gene targets. A prediction is proposed using bioinformatics tool within the interval [40..70](see sRNATarBase). FnrS is a sRNA of 122 nucleotides in length. It is produced by the bacterium in anaerobic conditions under the control of the FNR and ArcA proteins. This sRNA may regulate more than 30 mRNAs, mainly involved in energy metabolism [34]. For example, FnrS is involved in the regulation of enzymes required in the shift from aerobic to anaerobic conditions when the cell is undergoing an oxidative stress [35]. Two interacting region seeds have been identified using mutation experiments, from the positions 4 to 6 (when interacting with folE, folX and sodB) and from the positions 47 to 49 (when interacting with maE and gpmA)[35].

To illustrate the usefulness of *rNAV 2.0*, we exploited new experimental results that have been performed for both sRNAs. For each sRNA, a compilation of RNA-SEQ experiments provided a list of putative gene targets whose expression is related to the sRNA. To demonstrate if the interaction between the mRNA and sRNA is direct or indirect, further experimental works have to be done. Then, our objective is to prioritize the best candidates for designing this experimental work to validate the interaction. We carried out two types of analysis, a *Bottom Up* and a *Top Down* analysis. In the RyhB analysis, we started from the whole network, then made use of known biological constraints and translate them into filtration stages and at last we focused on the intersection with the putative targets. By contrast, for the FnrS analysis, we first looked at the putative targets (given by experimental works) to then apply different annotation filtering stages.

### Analysis of RyhB targets

**Fig. 3:**
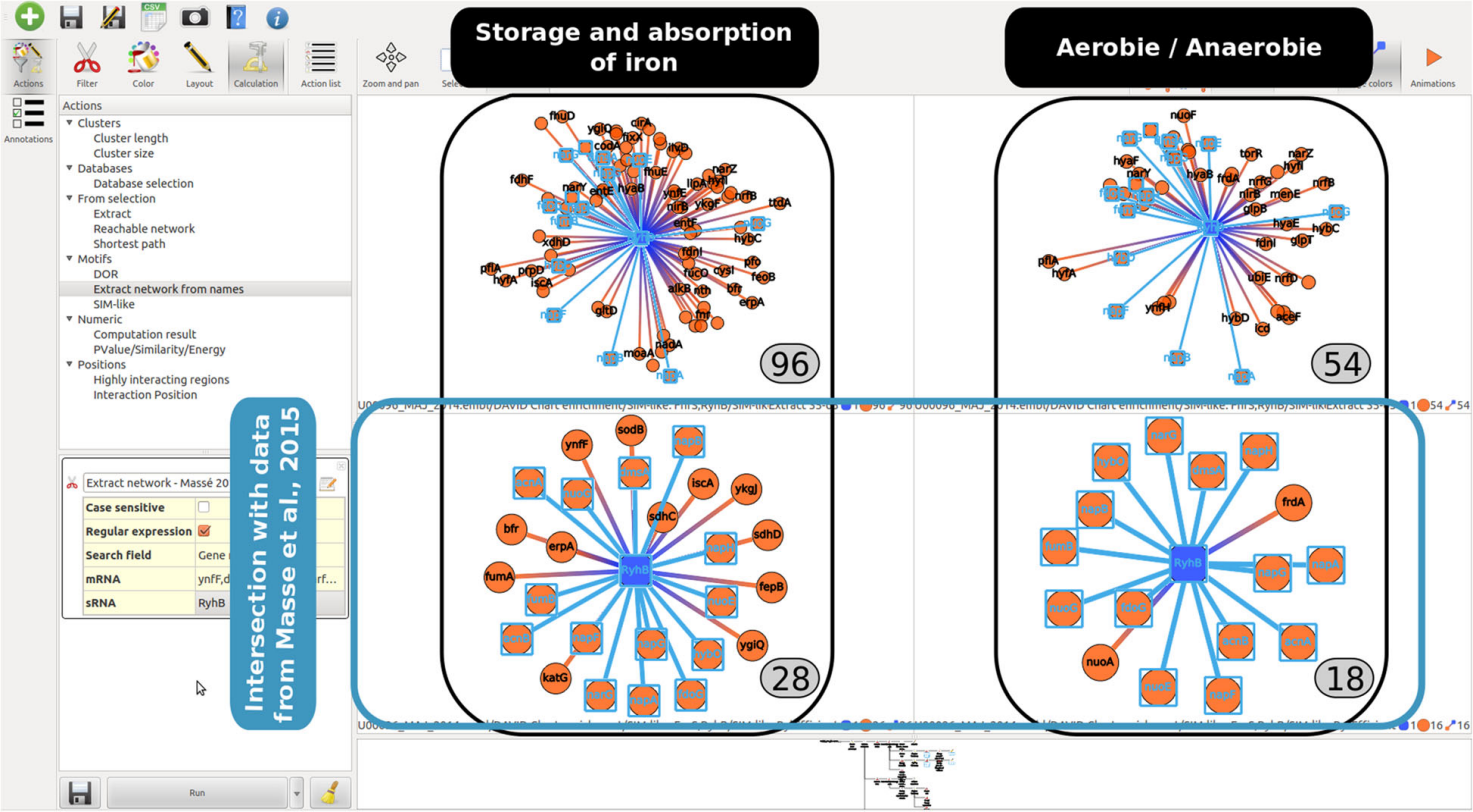
Exploration of RyhB regulated network. Starting from the complete RyhB network, the two sub-networks on the first line focus on targets that interact with the conserved region and involve specific annotations. Starting from the RyhB sub-network that depicts the targets of publication of [36], the two sub-networks on the second line depict a focus of targets enriched with annotations related to the iron storage and absorption, and to the presence or absence of oxygen. The common targets with both annotations are highlighted in *blue*, and this selection is propagated to the other screens. A layout algorithm has been applied to the latter panels

The following stages of this analysis are represented by the exploration tree view of *rNAV*: they correspond to the two first branches in the Fig. 2 and the resulting sub-networks are given in the Fig. 3. Using MS2-affinity purification coupled with RNA sequencing technology, 37 genes have shown a repression profile related to the expression of RyhB [36]. Starting from the complete RyhB SIM-like motif where more that 1000 targets were predicted by IntaRNA, we applied two consecutive filtering steps to filter out the prediction (i) with an IntaRNA score lower to –5 and (ii) with an interaction which overlaps the [35–65] sRNA region. Then, two subnetworks were extracted for showing up the targets with annotations related to (i) the storage and absorption of iron and to (ii) the presence or absence of oxygen. Respectively, we get 96 and 54 targets among which 28 and 18 were included in the data given by Masse et al. [36].

At last, considering the intersection of both filtering stages, 15 targets are common and may be of particular interest for designing experimental protocols with the objective to identify new targets that are both involved in the iron storage/absorption and in the aerobic/anaerobic energy metabolism.

### Analysis of FnrS targets

In a first work dedicated to the study of FnrS [35], the use of an experimental protocol to over-express this sRNA has demonstrated at least 2-fold decreasing expressions of 32 genes. In a second work of Boysen et al. [37], global proteomic and transcriptomic approaches were combined. Using a threshold of 1,5 fold time of under expression, a list of 16 regulated putative targets was proposed. After the subtraction of the 13 known targets, the total given by these two publications and for which a prediction was given by IntaRNA put the number of putative targets to 18.

**Fig. 4:**
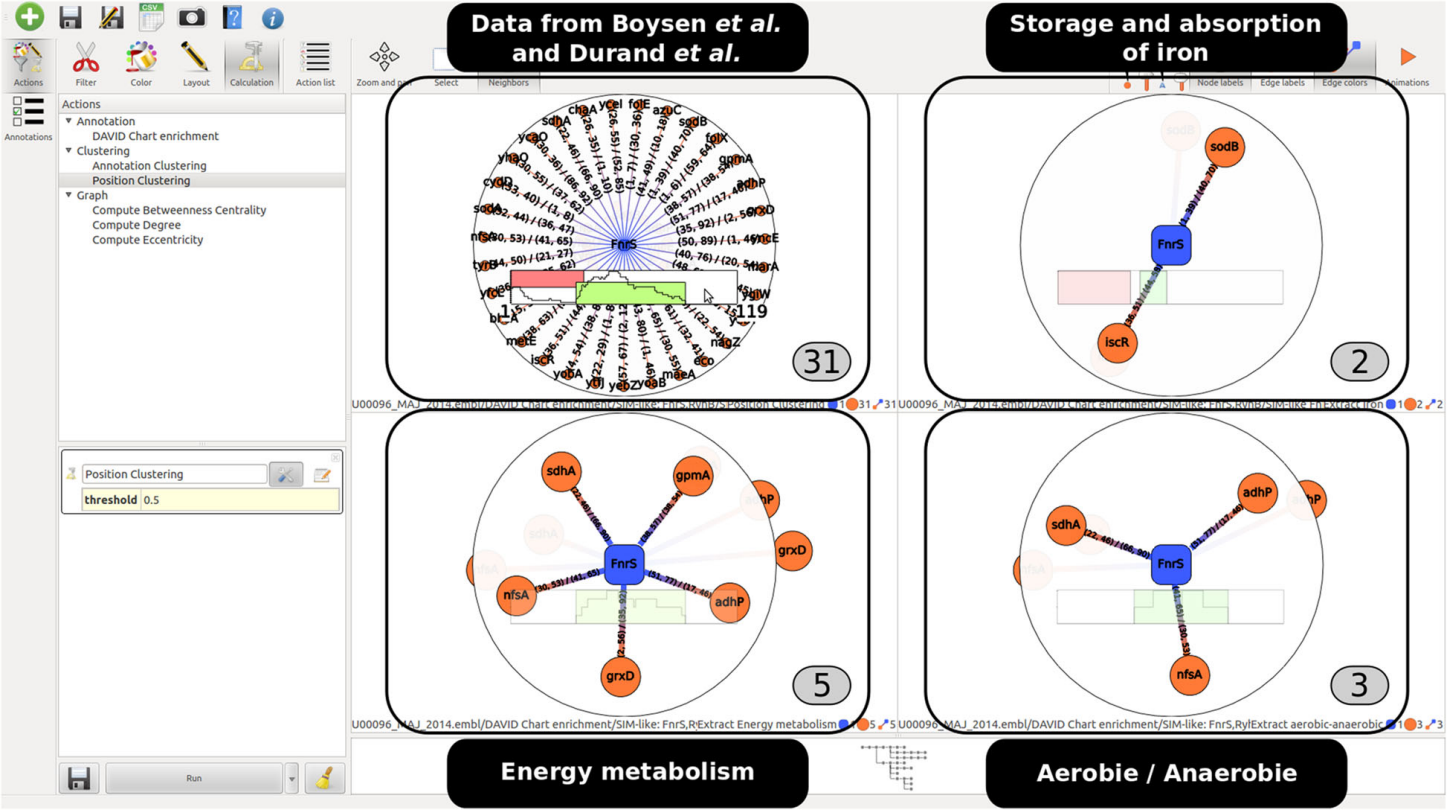
Exploration of FnrS regulated network. Starting from the FnrS network, The first sub-network depicts the results given by the experimental works of [35, 37] and the 13 validated targets. In the first display, the colored rectangles in *red* and *green* represent the two interaction position clusters given by the MCL algorithm. The three other displays depict respectively the targets related to the iron storage and absorption, to the energy metabolism and to the aerobic/anaerobic keywords. The interaction region cluster involved by the enriched targets is displayed under the three subnetworks

Focusing on these data from the beginning of the analysis, the following stages correspond to the third and fourth branches of the exploration tree in the Fig. 2 and the resulting sub-networks are given in the Fig. 4. First, we used the MCL algorithm to cluster together the 31 targets according their interaction region. We obtained two region clusters which contain the two interaction seeds that were experimentally proved. Then, this position information was used in combination with the annotation after the application of a DAVID enrichment analysis. Three filtering steps were applied, using annotations related to the iron storage and absorption, the energy metabolism and the presence and absence of oxygen. The results are given as sub-networks in the Fig. 4. From the list of 31 genes, we highlighted three new targets (adhP, grxD, and nfsA) that satisfy biological constraints common to validated experimental works.

## Results and discussion

We performed an analytical pipeline based on the sRNA-mediated regulatory network of *Escherichia coli* with the two sRNAs, FnrS and RyhB.

First, RyhB, whose expression is induced by a low level of iron, is known to interact with iron-containing enzymes and iron-transporting proteins [30]. From the 37 repressed genes given by [36], we have highlighted using *rNAV* a list of 15 targets. Making use of this small list of genes, we have carried out a deeper study to prioritize few genes according to a coordinated regulation hypothesis. For instance:

- Three targets are components of the NADH:quinone oxidoreductase I (nuoA, nuoE and nuoG) and may required a coordinated regulation (SIM-like motif).
- Four other targets are parts of the nap operon related to the periplasmic nitrate reductase (napF, napG, napA, and napH). Moreover, Kern et al. [38] have studied in *Wolinella succinogenes* the membrane-bound NapGHF complex and have shown its multi-functional role in the maturation of napA precursor. A common regulation of these proteins may be of interest to demonstrate their related actions with the periplasmic nitrate reductase napA of *Escherichia coli* which plays an important role during the anaerobic growth in low-nitrate environments [39].
- The two Fumarase A and B (fumA and fumB) are also parts of the targets and are two of the three fumarase isozymes participating in the TCA cycle. It has been shown that the expression of each of these two isozymes was maximum in aerobic or anaerobic conditions [40].

**Fig. 5:**
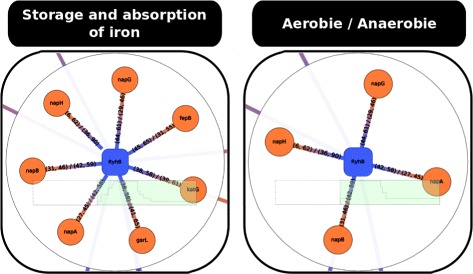
Two sub-networks corresponding to new targets from [41] that are enriched with annotations related to the iron storage and absorption and that interact with the conserved region proposed by *rNAV*

Interestingly, all of these new targets have also been identified using ribosome profiling (Ribo-seq) in a recent work demonstrating the new great applications of these experimental approaches for the genome-scale identification of sRNA targets [41]. Using the same filtering steps (score and region) and according to the annotations related to (i) the storage and absorption of iron and to (ii) the presence or absence of oxygen, we have obtained two lists of 7 and 4 targets (coming resp. from the lists of 96 and 54 targets). Of particular interest, the nap operons are retrieved for both annotation filtering stages (see Fig. 5).

Second, FnrS is involved in the regulation of enzymes required in the aerobic-anaerobic transition when the cell is undergoing an oxidative stress [35]. Of particular interest for this sRNA, two interaction regions are known from the 13 validated targets. With *rNAV* and using the MCL algorithm for the 31 targets given by [35, 37], this two regions have been perfectly retrieved. When using functional information known for FnrS, only 7 targets are proposed - with 4 targets that were already known (see Table 1). While these two seed regions are involved in iron storage, only the second one interacts with genes related to the aerobic/anaerobic energy metabolism. This observation may be of interest to investigate the reasons for the presence of two interaction regions.

In both studies, the combination of additional biological features have given compelling arguments for prioritizing candidates. Moreover, *rNAV* improved our exploration of the sRNA-mediated networks. Very quickly, *rNAV* can help to realize a deep study that seems very complex at first. This advantage is illustrated by the very small number of steps that were designed in *rNAV* (see the *rNAV* pipelines in the Fig. 2) for investigating the RyhB and FnrS targets.

## Conclusions

To address the large amounts of data generated by new sequencing technologies, the integration of additional biological information is of great importance. Focusing on sRNA-mediated networks, bioinformatics tools are necessary to prioritize regulated targets but are still producing a prohibitive number of candidates. To address this problem, our strategy relies on providing *a posteriori* multipurpose information, according to the heterogeneous data extracted from databases and the visual exploration/analysis of the biological network. We present a new software for developing bioinformatics strategies to explore sRNA-mediated networks. The methodology used in *rNAV* combines bioinformatics tools which predict interactions and visualization techniques, and is freely available at http://rnav.labri.fr.

## Availability and requirements

*rNAV 2.0* is free software licensed under LGPL. Source code is available at http://rnav.labri.fr. rNAV was done within the EVIDEN and the MycoRNA projects. rNAV has been developed in C++ and python and is available for linux OS under LGPL.

## Additional files

Additional file 1 Feature comparison. Comparison between the two versions of rNAV with Cytoscape and dedicated plugins. (PDF 53 kb)

Additional file 2 Data description. Data description and availability, and parameter settings used in this study. (PDF 101 kb)

## Abbreviations

MCL: Markov chain clustering
mRNA: Messenger Rubonucleic acid
RNA: Rubonucleic acid
sRNA: Small Rubonucleic acid
