# Supplementary material for: rNAV 2.0: a visualization tool for bacterial sRNA-mediated regulatory networks mining
Source: BMC Bioinformatics. 2017 Mar 23;18:188. doi: 10.1186/s12859-017-1598-8 (PMC5364647; doi:10.1186/s12859-017-1598-8)
Supplement: Supplementary file 1 — Feature comparison. Comparison between the two versions of rNAV with Cytoscape and dedicated plugins. (PDF 53 kb) [file 12859_2017_1598_MOESM1_ESM.pdf]

Data material

In the following, we give further explanations about the filtering steps used to perform the analysis of the case study.

1- The sRNA-mediated regulatory network of *Escherichia coli*

More than 80 small RNAs have been experimentally identified within *Escherichia coli* including a large set of sRNAs that are playing regulatory functions, by base-pairing 5' UTR mRNA regions. In this context, we performed an analytical strategy based on the sRNA-mediated regulatory network with the two sRNAs, FnrS and RyhB, which are both multi-targeting mRNAs with validated common targets.

2- The construction of the network

We constructed the network using rNAV derived from both :

- A sRNA multi-fasta file
  - **FnrS** (**ecocyc accession number: G0-8872**) is involved in the regulation of enzymes required in the aerobic-anaerobic transition when the cell is undergoing an oxidative stress (Durand *et al,* 2010).

```
>fnrS
GCAGGTGAATGCAACGTCAAGCGATGGGCGTTGCGCTCCATATTGTCTTACTTCCTTTTTTGAATTACT
GCATAGCACAATTGATTCGTACGACGCCGACTTTGATGAGTCGGCTTTTTTTT
```

  - **Ryhb** (**ecocyc accession number: G0-10677**): its expression is induced by a low level of iron, is known to interact with iron-containing enzymes and iron transport and storage proteins (Massé *et al,* 2007).

```
>ryhB
GCGATCAGGAAGACCCTCGCGGAGAACCTGAAAGCACGACATTGCTCACATTGCTTCCAGTATTACTTA
GCCAGCCGGGTGCTGGCTTTT
```

- An EMBL file for genome of *Escherichia coli* (**Accession number: U00096**), using adjustable parameters for the 5' UTR extraction (as parameters, we extracted regions located 70/+5 from the AUG start codon) of each mRNA sequences.

Then, interactions were computed with IntaRNA (seed = 6) through rNAV and the enrichment was performed using the DAVID web-services.
