# Supplementary material for: rNAV 2.0: a visualization tool for bacterial sRNA-mediated regulatory networks mining
Source: BMC Bioinformatics. 2017 Mar 23;18:188. doi: 10.1186/s12859-017-1598-8 (PMC5364647; doi:10.1186/s12859-017-1598-8)
Supplement: Supplementary file 2 — Data description. Data description and availability, and parameter settings used in this study. (PDF 101 kb) [file 12859_2017_1598_MOESM2_ESM.pdf]

			RNAV 1.0	RNAV 2.0	CytoScape
**Visualization**		Node-link diagram	-	-	-
		Exploration tree view	-	-	**N.A.**
		multiple linked views	-	-	-
		interacting regions of an RNA	-	-	**N.A.**
**Interaction tools**		Box-selection	-	-	-
		Zoom-and-pan	-	-	-
		Dedicated Neighborhood:			
		Neighborhood highlight	-	-	**N.A.**
		RNA region selection	-	-	**N.A.**
**Algorithms**	**Layout**	Dedicated layered	-	**N.A.**	-
		Circular	-	-	-
		Force-directed	-	-	-
		(Fast) Multiscale force-directed	-	-	-
	**Filter**	RNAs from their clusters according to			
		Cluster interaction region length	**N.A.**	-	**N.A.**
		Number of interactions within the cluster	**N.A.**	-	**N.A.**
		Database annotations	-	-	-
		From a selection :			
		Extract (create a subgraph)	-	-	-
		Reachable network	-	-	-
		Shortest path	-	-	-
		Motifs :			
		DOR	**N.A.**	-	**N.A.**
		SIM-like	**N.A.**	-	**N.A.**
		Extract from RNA name	-	-	-
		According to numerical values :			
		Pvalue/Similarity/Energy	-	-	-
		Computed metric	-	-	-
		Interaction region	-	-	**N.A.**
	**Coloration**	Edges :			
		Pvalue/Similarity/Energy	**Pvalue**	-	-
		Nodes :			
		Type of RNA	-	-	-
		Node degree	-	-	-
		Node eccentricty	-	-	-
		Node betweenness centrality	-	-	-
	**Computation**	Interaction clustering for each RNA based on :			
		Annotations	**N.A.**	-	**N.A.**
		Interacting regions	-	-	**N.A.**
		Metrics :			
		Degree	-	-	-
		Eccentricty	-	-	-
		Betweenness centrality	-	-	-
		Enrichment analysis	**N.A.**	-	-
**Input Data**	**Data format**	rNAV files	-	-	**Not Applicable**
		CSV files (for nodes and edges)	-	-	-
		Genome (embl) & Fasta (sRNA sequences)	**N.A.**	-	-
	**Prediction tools**	Ssearch	**N.A.**	-	
		IntaRNA	**N.A.**	-	
**Other Features**	**Rendering Parameters**	Show/Hide node and edge labels	-	-	-
		Select property to be displayed as labels	-	-	-
		Change label sizes	-	-	-
		Change node sizes	-	-	-
		Enable/Disable animation during transitions	**N.A.**	-	**N.A.**
	**Algorithm Pipelines**	Edit/save/Re-use algorithm pipelines	**N.A.**	-	**N.A.**

List of the main features supported by rNAV 2.0 in comparison to its prototypical version (rNAV 1.0) and, to Cytoscape [11] and its available plugins (*e.g.* ReNE[12], Bingo[13] or ClueGO[14]). In this table, N.A. stands for Not Available.
